# Targeted sequencing may facilitate differential diagnostics of pulmonary tumours: a case series

**DOI:** 10.1186/s13000-017-0621-8

**Published:** 2017-03-27

**Authors:** Kajsa Ericson-Lindquist, Anna Johansson, Per Levéen, Göran Elmberger, Göran Jönsson, Johan Staaf, Hans Brunnström

**Affiliations:** 1 Department of Pathology, Regional Laboratories Region Skåne, SE-221 85 Lund, Sweden; 2 Department of Pathology, Örebro University Hospital, SE-701 85 Örebro, Sweden; 3 Department of Clinical Sciences in Lund, Division of Oncology and Pathology, Lund University, SE-221 00 Lund, Sweden

**Keywords:** Lung cancer, Metastasis, NGS, Pyrosequencing, Synchronous

## Abstract

**Background:**

Histopathological diagnosis is important for prognostication and choice of treatment in patients with cancer in the lung. Metastases to the lungs are common and need to be distinguished from primary lung cancer. Furthermore, cases with synchronous or metachronous primary lung cancers (although infrequent) are often handled differently than cases with lung cancer with intrapulmonary metastasis or relapse, respectively. In some cases, morphology and immunohistochemical staining is not sufficient for certain diagnosis.

**Methods:**

The present study included six cases where molecular genetic analysis in form of pyrosequencing or targeted next-generation sequencing was of value for certain diagnosis of selected tumours in the lung.

**Results:**

Two of the included cases were rare metastases to the lung; colorectal cancer with IHC profile consistent with primary lung cancer and malignant adenomyoepithelioma of the breast, respectively, where molecular genetic analysis was of aid for proving the relationship to the primary tumour. The other four cases were multiple lung adenocarcinomas where molecular genetic analysis was of aid to distinguish between intrapulmonary metastasis and synchronous tumour.

**Conclusions:**

Comparison of molecular genetic profile may be an important tool for determination of relationship between tumours in some situations and should always be considered in unclear cases. Further studies on concordance and discordance of molecular genetic profiles between spatially or temporally different tumours with common origin may be helpful for improved diagnostics of pulmonary tumours.

## Background

Histopathological diagnosis is important for choice of treatment in patients with cancer in the lung. Metastases to the lungs are common and need to be distinguished from primary lung cancer, and the treatment of primary lung cancer is dependent on histopathological type [1–3]. Furthermore, cases with synchronous or metachronous primary lung cancers (although infrequent [4, 5]) are often handled differently than cases with lung cancer with intrapulmonary metastasis or relapse, respectively.

The basis for histopathological diagnosis is morphology, with the addition of immunohistochemical (IHC) staining when needed. For example, a limited panel of thyroid transcription factor 1 (TTF-1) in combination with estrogen receptor and GATA3 or with cytokeratin (CK) 7 and CK20 normally separates lung cancer from breast cancer and colorectal cancer, respectively, with high accuracy [6–10]. However, sometimes the morphological appearance may be indistinct and the results of IHC staining may differ from the typical, with for example positive GATA3 reported in 8% of lung adenocarcinomas [11–16]. Thus, there is a need for additional diagnostic analyses or markers, at least in some cases.

It may also be difficult to separate synchronous or metachronous primary lung cancers from intrapulmonary metastasis or relapse, respectively. According to the TNM staging classification and guidelines from the American College of Chest Physicians (ACCP), separate tumours of different histopathological type are to be considered synchronous/metachronous [17, 18]. In a case with multiple tumours of the same histopathological type, the TNM classification underscores the pathologist’s opinion based on differences in morphology, IHC and molecular genetic characteristics and, in case of squamous cell carcinoma, association with carcinoma in situ for consideration of synchronous/metachronous primary lung cancer [18]. The ACCP guidelines also acknowledge different molecular genetic characteristics and association with carcinoma in situ (though not limited to only squamous cell carcinomas), but emphasize a multidisciplinary approach and that the tumours should be in separate lobes and not appear within 2 years, as suggested in the early study by Martini and Melamed [17, 19, 20]. According to both guidelines, nodal (mediastinal or with common drainage) or distant metastases should not be present in synchronous primary lung cancers.

Molecular genetic analysis is normally used for treatment prediction (such as identification of epidermal growth factor receptor [EGFR] mutations). As mentioned, it is also a recognised tool for distinguishing synchronous primary lung cancer from intrapulmonary metastasis, and its use in that respect has been reported already from several years ago, [21] although in our experience underused in the clinical setting. Single gene assays, which may be sufficient for treatment prediction today, may be of limited use in the differential diagnostics of synchronous tumour vs. intrapulmonary metastasis as most lung adenocarcinomas are not *EGFR* mutated. However, next generation sequencing (NGS), today implemented at many sites, should be of more value for comparison of genetic profiles, especially targeted NGS which is more practical in the clinical setting than whole exome sequencing (WES), although the latter may be performed on formalin-fixed paraffin-embedded (FFPE) tissue as well [22].

Significant mutational intra-tumour heterogeneity and discordance between primary tumour and metastasis as well as temporal differences during treatment has been reported in for example renal and ovarian cancer [23–25]. However, in these studies with extensive sequencing some ubiquitous mutations were commonly found in all spatially or temporally different clones. Furthermore, a high or perfect concordance between primary tumour and metastasis has been seen in colorectal and lung cancer and between areas with different growth patterns within lung adenocarcinomas when looking at common driver mutations such as *EGFR*, *KRAS*, *NRAS* and *BRAF* [26–30].

In the present study, we present six cases where molecular genetic analysis aided in the diagnostics of pulmonary tumours. With this case series, we aim to highlight the topic of comparison of molecular genetic profiles between two or more tumours as a diagnostic tool in an accessible way that also is easy to relate to in the clinical setting.

## Methods

### Patient selection and investigation

The present study included selected tumours of interest from lung resections where molecular genetic analysis had been an aid for definite diagnosis. All cases were surgically treated for one or more lung tumours at the Skåne University Hospital, Lund, Sweden during the years 2011–2016.

The routine investigational work-up included computed tomography (CT), positron emission tomography with CT and bronchoscopy including endobronchial ultrasound (EBUS) guided fine needle aspirations from hilar and mediastinal lymph nodes. In some cases, transthoracic core needle biopsy was also performed. After surgical resection the lung specimen were fixed in 10% neutral aqueous formalin (corresponding to 4% formaldehyde) for typically 48 h before gross sectioning and further processing before paraffin-embedding. The routine sampling included embedding of all tumour if ≤2 cm in size and at least 1 section per cm (i.e. at least 3 if 2.1-3.0 cm) often including one super mega cassette if more than 2 cm in size.

### Immunohistochemical staining

Various IHC stains of relevance were performed in the clinical setting at the Dept. of Pathology, Lund, Region Skåne, Sweden, as part of the histopathological diagnostic procedure. Four micrometer thick sections from FFPE tissue blocks were pre-treated and stained in a Ventana Bench-Mark Ultra using Ventana ultraView Universal DAB Detection Kit (Ventana Medical Systems, Tucson, AZ). Recommended control tissue was used on each slide. The clones and vendors for the antibodies included in this study were TTF-1 clone 8G7G3/1, CK7 clone OV-TL 12/30, CK20 clone SP33, CDX2 clone EPR2764Y, S100 polyclonal, estrogen receptor clone SP1, progesterone receptor clone 1E2, HER2 clone 4B5, ALK clone D5F3, all Ventana Medical Systems (Tucson, AZ), napsin A clone IP64, CK5 clone XM26, both Novocastra/Leica Biosystems (Kista, Sweden), p63 clone 4A4, smooth muscle specific actin clone 1A4, both Dako (Glostrup, Denmark), p40 clone BC28, Histolab Products/Biocare Medical (Gothenburg, Sweden), and GATA3 clone L50-823, Cell Marque (Rocklin, CA).

### Fluorescence in situ hybridization (FISH)

FISH for *ALK* gene rearrangements was performed in the clinical setting at the Dept. of Pathology, Lund, Region Skåne, Sweden. During the studied time period, FISH was initially the only method for *ALK* analysis in the routine practice, but was later only used as complement if ALK IHC staining was unclear. The Vysis Break Apart probe (Abbott Molecular, Abbott Park, IL) was used according to the manufacturer's protocol. Split red and green signals or isolated red signal in ≥15% of at least 50 nuclei was considered positive (in practice, normally >100 tumour cell nuclei were evaluated).

### Molecular genetic analysis

Molecular genetic analysis, in form of pyrosequencing and targeted NGS, was performed at the Dept. of Clinical Sciences Lund/Dept. of Pathology, Lund, Region Skåne, Sweden. The analyses were previously validated for routine clinical diagnostics within the department. For each sample, a representative area with high frequency of viable malignant cells (at least 10%) was identified by a pathologist on a hematoxylin-eosin (HE) stained slide from a FFPE tissue block. Six five-micrometer thick sections were then taken for molecular analysis followed by a new HE stained section to ensure the material being representative.

### Pyrosequencing

For single gene analysis (routine analysis in Region Skåne before 2015), DNA was extracted using the QIAamp DNA FFPE Tissue Kit for paraffin embedded tissues according to the manufacturer's protocol (Qiagen, Hilden, Germany), except that the proteinase K digestion was extended to overnight. Mutational status for hotspot mutations in *EGFR* and *KRAS* were obtained using pyrosequencing (PyroMark Q24 sequencer, TheraScreen EGFR Pyro Kit and TheraScreen KRAS Pyro Kit, DxS/Qiagen, Hilden, Germany) according to the manufacturer's protocol.

### Targeted NGS

For targeted NGS, DNA was extracted using the Qiagen AllPrep kit (Qiagen, Hilden, Germany) for FFPE tissue and automated on the QIAcube instrument (Qiagen) according to the manufacturer's protocol, except that the proteinase K digestion was extended to overnight. The NGS-based mutation analysis was performed using the Illumina TruSight Tumor gene panel on a MiSeq instrument according to manufacturer’s instructions (Illumina, San Diego, CA, US). Analysed regions included a selected set of complete exons in 26 genes: *AKT1* (exon 2), *ALK* (exon 23), *APC* (exon 15), *BRAF* (exons 11, 15), *CDH1* (exons 8, 9, 12), *CTNNB1* (exon 2), *EGFR* (exons 18, 19, 20, 21), *ERBB2* (exon 20), *FBXW7* (exons 7, 8, 9, 10, 11), *FGFR2* (exon 6), *FOXL2* (exon 1), *GNAQ* (exons 4, 5, 6), *GNAS* (exons 6, 8), KIT (exons 9, 11, 13, 17, 18), *KRAS* (exons 1, 2, 3, 4), *MAP2K1* (exon 2), *MET* (exons 1, 4, 13, 15, 16, 17, 18, 20), *MSH6* (exon 5), *NRAS* (exons 1, 2, 3, 4), *PDGFRA* (exons 11, 13, 17), *PIK3CA* (exons 1, 2, 7, 9, 20), *PTEN* (exons 1, 2, 3, 4, 5, 6, 7, 9), *SMAD4* (exons 8, 11), *SRC* (exon 10), *STK11* (exons 1, 4, 6, 8), and *TP53* (exons 2, 3, 4, 5, 6, 7, 8, 9, 10, 11). Prior to library preparation a quality control assay was performed as described in the TruSight Tumor instructions. Alignment, quality filtering, variant calling, and variant annotation were performed using the standard MiSeq Reporter and VariantStudio analysis pipeline (Illumina). Only nonsynonymous variants with a quality score equal to 100 that passed the bi-directional sequencing quality filter in TruSight Tumor were considered. Non-targetable mutations with a frequency of <3% were not routinely reported.

Due to change of platform in the clinical setting, for two cases (no. 5 and 6) the NGS-based mutation analysis was instead performed using the Ion Ampliseq Cancer Hotspot panel v2 on an Ion Torrent PGM instrument according to manufacturer’s instructions (Thermo Fisher Scientific, Waltham, MA, US). Analysed regions included a selected set of complete exons in 50 genes: *ABL1* (exon 4–7), *AKT1* (exon 3, 6), *ALK* (exon 23, 25), *APC* (exon 16), *ATM* (exon 8, 9, 12, 17, 26, 34, 35, 36, 39, 50, 54–56, 59, 61, 63), *BRAF* (exon 11, 15), *CDH1* (exon 3, 8, 9), *CDKNA2* (exon 2), *CSF1R* (exon 7, 22), *CTNNB1* (exon 3), *EGFR* (exon 3, 7, 15, 18–21), *ERBB2* (exon 19–21), *ERBB4* (exon 3, 4, 6–9, 15, 23), *EZH2* (exon 16), *FBXW7* (exon 5, 8–11), *FGFR1* (exon 5, 8), *FGFR2* (exon 7, 9, 12), *FGFR3* (exon 7, 9, 14, 16, 18), *FLT3* (exon 11, 14, 16, 20), *GNA11* (exon 5), *GNAQ* (exon 5), *GNAS* (exon 8, 9), *HNF1A* (exon 3, 4), *HRAS* (exon 2, 3), *IDH1* (exon 4), *IDH2* (exon 4), *JAK2* (exon 14), *JAK3* (exon 4, 13, 16), *KDR* (exon 6, 7, 11, 19, 21, 26, 27, 30), *KIT* (exon 2, 9–11, 13–15, 17, 18), *KRAS* (exon 2–4), *MET* (exon 2, 11, 14, 16, 19), *MLH1* (exon 12), *MPL* (exon 10), *NOTCH1* (exon 26, 27, 34), *NPM1* (exon 11), *NRAS* (exon 2–4), *PDGFRA* (exon 12, 14, 15, 18), *PIK3CA* (exon 2, 5, 7, 8, 10, 14, 19, 21), *PTEN* (exon 1, 3, 5–8), *PTPN11* (exon 3, 13), *RB1* (exon 4, 6, 10, 11, 14, 17, 18, 20, 21, 22), *RET* (exon 10, 11, 13, 15, 16), *SMAD4* (exon 3–6, 8–12), *SMARCB1* (exon 2, 4, 5, 9), *SMO* (exon 3, 5, 6, 9, 11), *SRC* (exon 14), *STK11* (exon 1, 4, 5, 6, 8), *TP53* (exon 2, 4–7, 10), *VHL* (exon 1–3). Alignment, quality filtering, variant calling, and variant annotation were performed using the standard Ion Reporter and Torrent Suite software (Thermo Fisher Scientific). Non-targetable mutations with a frequency of <5% were not routinely reported.

## Results

### Case 1

**Fig. 1:**
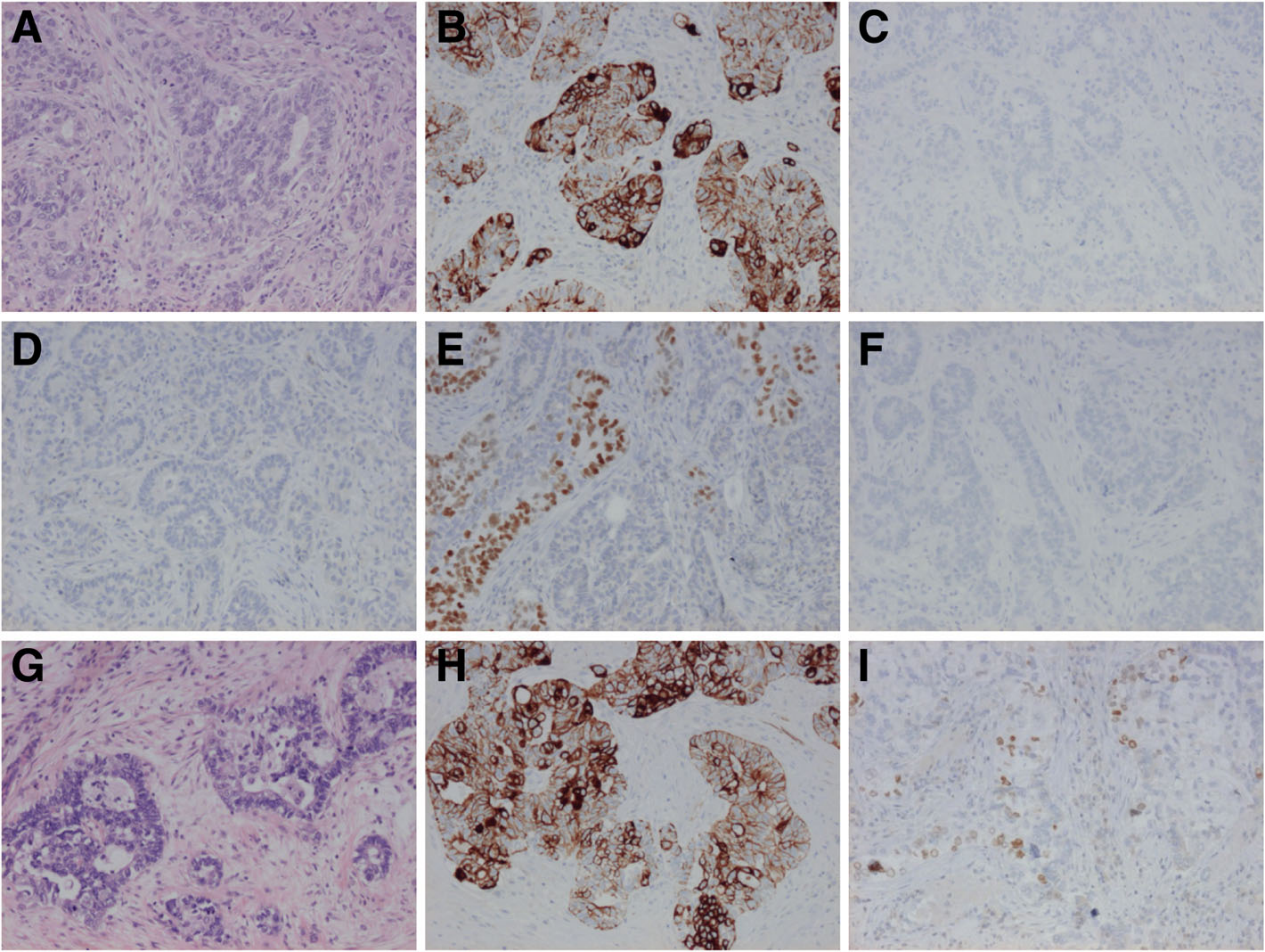
Case 1. Metastases of rectal cancer to the lung and liver with atypical immunohistochemical profile. **a**-**f** The metastasis to the lung. **a** HE, (**b**) CK7, (**c**) CK20, (**d**) CDX2, (**e**) TTF-1, (**f**) napsin A. **g**-**i** The metastasis to the liver. **g** HE, (**h**) CK7, (**i**) TTF-1. Note that CK20 and CDX2 were also negative in the metastasis to the liver (not shown). All images x10 objective

Case 1 was a 58 years old woman who was surgically treated for one tumour in the liver and one in the lung. Both were suspected to be metastases from a previous rectal cancer treated 3 years earlier. There was no evidence of lymph node involvement or other metastases. Both the lung and liver tumour were adenocarcinomas. The lung tumour was almost round, 1.5 cm in diameter, with central necrosis, and exhibited mostly solid but also partly cribriform growth and with a few separate glandular structures. The cells were cuboid or irregular to a greater extent than cylindrical. The liver tumour had similar morphology but somewhat more resembled colorectal cancer. Both the lung and liver tumour was diffusely positive for CK7 (>90% of the cells) and partially for TTF-1 (15% of the cells in the lung tumour and in 5% in the liver tumour). CK20, CDX2, and napsin A were completely negative. See Fig. 1.

The biopsy from the previous rectal cancer was reviewed, and IHC staining showed positive CK7 while CK20 and TTF-1 were both negative. The surgical specimen had the same profile, but there was very limited amount of tumour due to neoadjuvant radiotherapy. Clinico-radiologically metastasis of rectal cancer was suggested, but considering the IHC profile, a separate lung cancer with liver metastasis was proposed by the pathologist, especially since TTF-1 (clone 8G7G3/1) was positive in the lung and liver tumours, although the CK7/CK20 profile was the same in the rectal cancer.

Molecular analysis using pyrosequencing showed the same *KRAS* mutation (c.38G > A) in both the rectal cancer (tested on the biopsy) and the lung tumour, supporting that the lung and liver tumours were metastases of the rectal cancer. Analyses of *EGFR* (pyrosequencing) and *ALK* (FISH) were negative in the lung tumour. There was insufficient material from the primary tumour to enable a follow-up NGS analysis for comparison with the metastases, but a later confirmatory targeted NGS analysis of the liver metastasis showed the same *KRAS* mutation and also a *TP53* mutation (c.743G > A).

A second lung metastasis was surgically treated a year after the first. It had the same morphology as the previous lung tumour and was partly TTF-1 positive as well. Two years later, the patient presented with CNS symptoms, and a metastasis was found in the frontal lobe. The metastasis was TTF-1 negative, but otherwise the same IHC profile with positive CK7 and negative CK20 and CDX2.

### Case 2

**Fig. 2:**
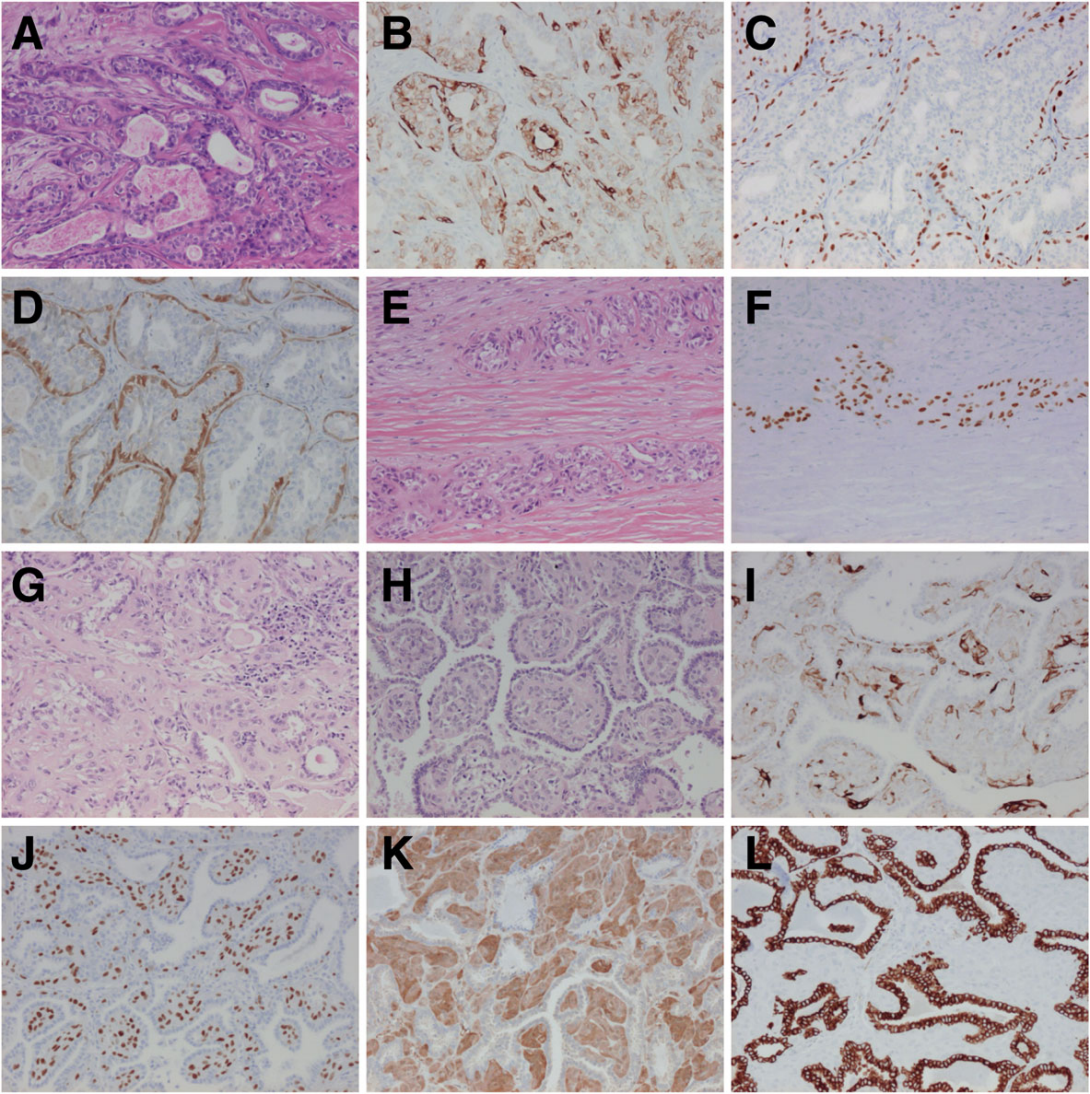
Case 2. Malignant adenomyoepithelioma in the breast with metastases to the lung. **a**-**f** The breast tumour. **a** HE, (**b**) CK5, (**c**) p40, (**d**) S100, (**e**) HE, peripheral part of the tumour suspicious for invasion, (**f**) p40, peripheral part of the tumour. **g**-**l** One of the metastases to the lung. **g** HE, central part of the tumour, (**h**) HE, peripheral part of the tumour, (**i**) CK5, (**j**) p40, (**k**) S100, (**l**) CK7. Note the CK7-positive reactive alveolar epithelium (positive also for TTF-1, not shown) leading to a papillary appearance at the periphery of the tumour. All images x10 objective.

Case 2 was a 70 years old woman with two histologically identical lung tumours in the right upper and lower lobes, respectively, both surgically removed with wedge resections. Both tumours were round and about 2 cm in diameter and rather well-circumscribed. A previous biopsy had suggested possible squamous cell carcinoma, but the morphology and IHC profile with >90% of the cells positive for p40, S100 and smooth muscle-specific actin (CK5 was positive in about 40% of the cells) was consistent with epithelial-myoepithelial carcinoma. See Fig. 2. Based on the tumours’ macroscopic appearance and peripheral location in the lung it was concluded the tumours were metastases. There was no evidence of epithelial-myoepithelial carcinoma in the salivary glands or in accessory salivary glands in the bronchi.

The patient had a previous history of a breast tumour originally suggested to be at first hand consistent with ductal carcinoma in situ, approximately 2 cm in size, surgically treated almost 5 years earlier. The tumours from the lung and breast were reviewed by several pathologists in Sweden and one international expert without consensus regarding the breast tumour. The morphology of the peripheral cells in the breast tumour and the cells of the lung tumours was similar although the growth pattern when comparing with the breast tumour as a whole was not perfectly identical and there were different opinions among pathologists whether the breast tumour was invasive or not. However, additional IHC staining of the breast tumour showed positive CK5, p40, p63, S100 and smooth muscle actin in the basal and peripheral cells, while the luminal cells were partly positive for CK5 (about 15% of the luminal cells) and estrogen receptor (about 20%). See Fig. 2. Progesterone receptor and HER2 were negative. The lung tumours were negative for estrogen and progesterone receptor and HER2.

After targeted NGS analysis revealed the same *PIK3CA* mutation in both the breast and lung tumours there was a total agreement among the pathologists that the breast tumour was a malignant adenomyoepithelioma with metastases to the lung. In the breast there was also a *PTEN* mutation in a low frequency not seen in the metastases to the lung (checked for frequency less than 3%). There were no other mutations detected. See Table 1 for full NGS data. An initial FISH for *ALK* gene rearrangements was inconclusive in one of the metastasis in the lung, and further FISH analyses were not performed after the result of the targeted NGS.

**Table 1:** Data from targeted NGS for comparison of molecular profiles between tumours (note that there was not enough tissue from the primary tumour in case 1 for NGS analysis in addition to pyrosequencing)

Case no.	Tumour	Estimated tumour qty (%)	Run no.	Gene	Mutation frequency (%)	Transcript/HGVSc	Classification	Consequence	dbSNP ID
2	Breast tumour	30	ML00256	PIK3CA	36.3	NM_006218.2: c.3140A > G	Unknown significance	Missense variant	rs121913279
				PTEN	6.3	NM_000314.4: c.80-1G > C	Unknown significance	Splice acceptor variant	
	Lung tumour	40	ML00257	PIK3CA	24.8	NM_006218.2: c.3140A > G	Unknown significance	Missense variant	rs121913279
3	Upper lobe tumour	10	ML00279	KRAS	12.1	NM_033360.2: c.34G > T	Pathogenic	Missense variant	rs121913530
				CDH1	50.3	NM_004360.3: c.1774G > A	Unknown significance (probably germline variant)	Missense variant	rs35187787
				SMAD4	22.9	NM_005359.5: c.1495 T > C	Unknown significance	Missense variant	
3	Lower lobe tumour	10	ML00280	KRAS	4.8	NM_033360.2: c.34G > T	Pathogenic	Missense variant	rs121913530
				CDH1	51.2	NM_004360.3: c.1774G > A	Unknown significance (probably germline variant)	Missense variant	rs35187787
				SMAD4	6.3	NM_005359.5: c.1495 T > C	Unknown significance	Missense variant	
4	Right upper lobe tumour	25	ML00352	KRAS	27.5	NM_033360.3: c.183A > T	Pathogenic	Missense variant	rs17851045
4	Left upper lobe tumour	60	ML00329	EGFR	59.9	NM_005228.3: c.2235_2249delGGAATTAAGAGAAGC	Pathogenic	Missense variant, feature truncation	rs121913421
				TP53	24.5	NM_000546.5: c.473G > T	Presumed pathogenic	Missense variant	
4	Left lymph node metastasis	20	ML01103	EGFR	13.7	NM_005228.3: c.2235_2249delGGAATTAAGAGAAGC	Pathogenic	Missense variant, feature truncation	rs121913421
				TP53	13.7	NM_000546.5: c.473G > T	Presumed pathogenic	Missense variant	
5	Central tumour	15	366-16	BRAF	8.9	NM_004333: c.1406G > T	Pathogenic	Missense variant	rs121913355
				TP53	5.6	NM_000546: c.811G > A	Pathogenic	Missense variant	
5	Subpleural tumour	40	367-16	BRAF	37.6	NM_004333: c.1406G > T	Pathogenic	Missense variant	rs121913355
6	Right upper lobe tumour	35	330-16	EGFR	17.1	NM_005228: c.2573G > T	Pathogenic	Missense variant	rs121434568
				TP53	17.8	NM_000546: c.581 T > G	Pathogenic	Missense variant	
6	Right lower lobe tumour	40	329-16	(no mutations; ALK positive)					
6	Left lower lobe tumour	30	279-16	(no mutations; ALK positive)					

Exactly one year later one more metastasis, this time in the left lung, was surgically removed. It had the same morphological appearance as the previous metastases to the lung. The patient was still alive 18 months later with no evidence of any more metastases but with a suspicion of local relapse of the metastasis in the right upper lobe on a CT scan.

### Case 3

**Fig. 3:**
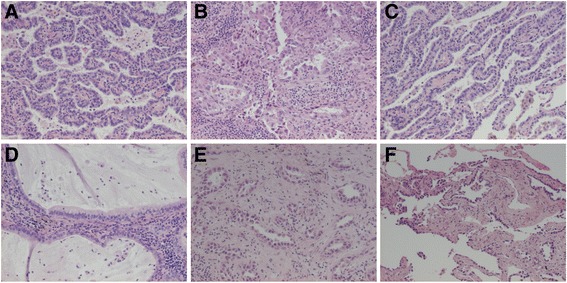
Case 3. Lung adenocarcinoma with intrapulmonary metastasis with partly different growth patterns but identical genetic profiles. **a**-**d** The tumour of the lower lobe with papillary (predominant), acinary, lepidic and mucinous (about 5%) growth patterns. **e**-**f** The tumour of the lower lobe with acinary (predominant) and lepidic growth patterns. All images HE and x10 objective

Case 3 was a 68 years old male with two rather small and slowly growing peripheral tumours in the lung, one in the right upper lobe (5 mm in size) and one in the right lower lobe (1.8 cm in size). There was no evidence of lymph node or distant metastases or malignant pleural exudate. Both tumours were surgically removed with wedge resections and were TTF-1 positive non-mucinous adenocarcinomas. The growth pattern was 50% papillary, 25% acinary, 20% lepidic and 5% mucinous (mostly lepidic) in the larger lower lobe tumour and 60% acinary and 40% lepidic in the upper lobe tumour. See Fig. 3. Both tumours exhibited some spreading through air spaces (STAS) and the tumour in the lower lobe also had pleural invasion but without any detected extension to the pleural surface (i.e. PL1). Furthermore, in the lower lobe there were a couple of additional, separate very small foci (<1 mm) of minimally invasive adenocarcinoma with lepidic and acinary growth, also detected during the surgical procedure.

The findings of the lower lobe was judged to be a tumour with metastasis within the same lobe (pT3), but it was discussed whether the tumour of the upper lobe represented a separate synchronous tumour (pT1a) or a metastasis (pT4). Targeted NGS analyses showed the same *KRAS* and *SMAD4* mutations in both the tumour of the upper lobe and one of the tumours in the lower lobe. See Table 1 for full NGS data. IHC staining for ALK was negative in both tumours. A later analysis of one of the very small tumours of the lower lobe (not of relevance for the clinical handling of the patient) revealed the same *KRAS* mutation while the *SMAD4* mutation was not seen.

Consequently, although the patient had a very small separate tumour with partly different growth pattern in the upper lobe and no lymph node or distant metastases, the case was considered to be a metastasis to another lobe (pT4) based on the identical molecular genetic profile. The patient suffered from severe renal insufficiency which limited the extent of surgical and chemotherapy treatment. However, the patient was still alive 18 months after surgery. At the time, a CT scan showed suspicious growth of a lung nodule and mediastinal lymph node enlargement, but EBUS-guided cytology was negative.

### Case 4

**Fig. 4:**
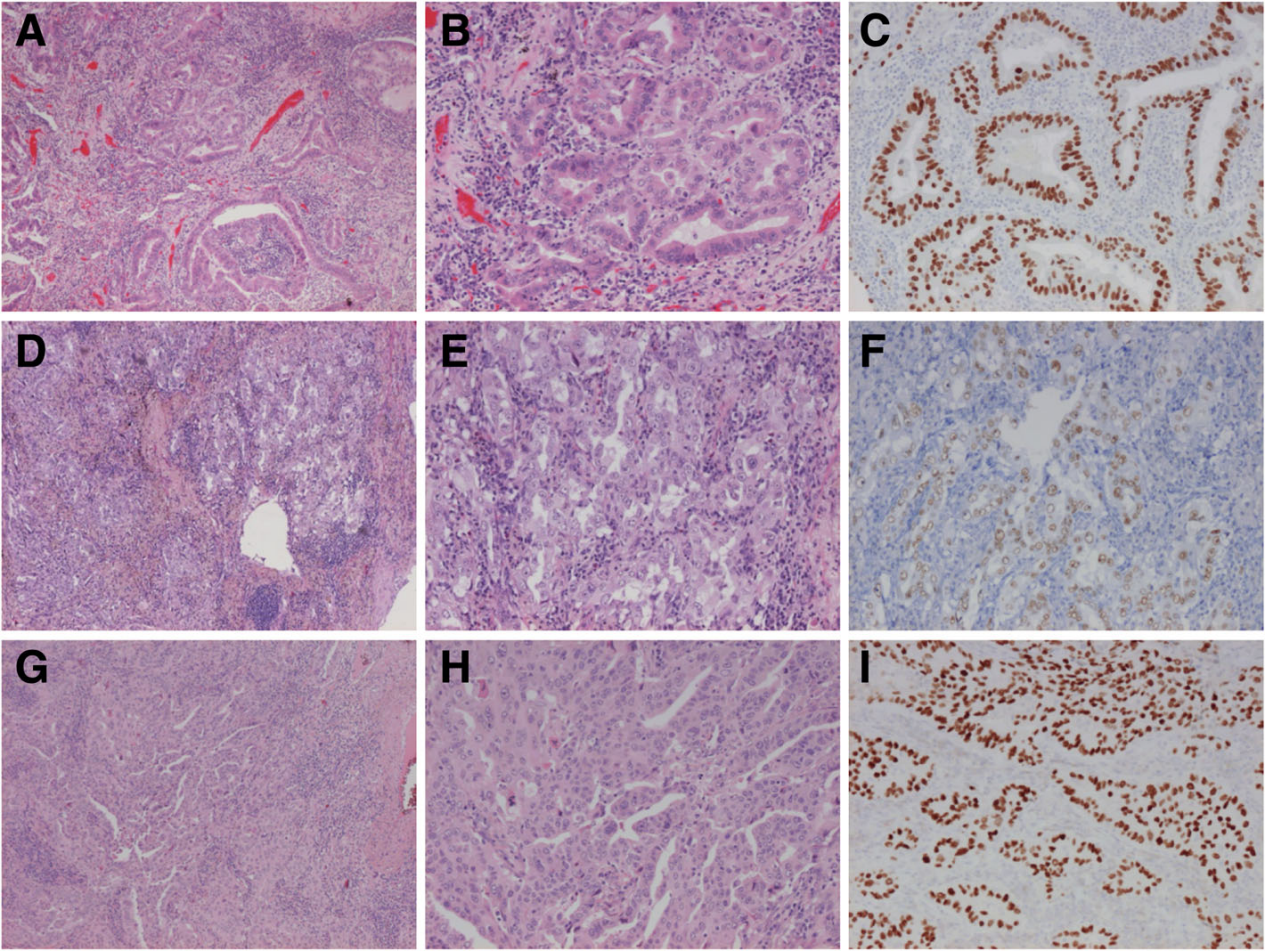
Case 4. Synchronous lung adenocarcinomas in upper lobes with similar morphology but different genetic profiles. **a**-**c** The *KRAS* mutated tumour in the right upper lobe. **a** HE, x4 objective, **b** HE, (**c**) TTF-1. **d**-**f** The *EGFR* mutated tumour in the left upper lobe. **d** HE, x4 objective, (**e**) HE, (**e**) TTF-1 (note the weaker TTF-1 may be due to suboptimal fixation). **g**-**i** Recurrence of the tumour in the left upper lobe. **g** HE, x4 objective, (**h**) HE, (**i**) TTF-1. All images x10 objective unless otherwise stated

Case 4 was a 64 years old woman with bilateral tumours in the upper lobes, both removed with wedge resections shortly after each other. The tumour in the left upper lobe was 2.5 cm in size and the one in the right upper lobe 1.9 cm. Both were TTF-1 positive non-mucinous adenocarcinomas. The growth pattern was 80% acinary, 15% solid and 5% cribriform (counting as acinary according to the WHO classification) in the tumour in the left lung, while the tumour in the right lung was 60% acinary, 30% cribriform and 10% solid. See Fig. 4. Although the two tumours were rather small and located in separate lungs and the patient had no evidence of lymph node or distant metastasis, it was decided by the multidisciplinary team to treat the patient as having metastatic disease (pT1b N0 M1a) since the histological type and growth pattern was identical. Thus, adjuvant chemotherapy was administered.

After 3 years there was a suspected local recurrence in the left upper lobe but no lymph node or distant metastases. Molecular genetic analysis was performed on the previously resected left upper lobe tumour with pyrosequencing showing an *EGFR* deletion in exon 19 (c.2235_2249del15) and negative IHC for ALK. A follow-up targeted NGS (being introduced at the time) instead detected a *KRAS* mutation in the tumour of the right upper lobe. The different profiles supported synchronous primary tumours (pT1b and pT1a). See Table 1 for full NGS data.

Since there was no evidence of lymph node or distant metastases, it was then decided to surgically remove the rest of the left upper lobe. The tumour was again a TTF-1-positive adenocarcinoma with mixed cribriform, acinary and solid growth. Targeted NGS detected the same *EGFR* deletion in exon 19 and also found a *TP53* mutation with a lower frequency. A later targeted NGS analysis confirmed the *EGFR* deletion in the original tumour of the left upper lobe but did not detect any *TP53* or other mutation.

One year later metastases to lymph nodes 4R and 4 L were confirmed on cytology from EBUS-guided fine needle aspirations. Again, a targeted NGS analysis confirmed the same *EGFR* deletion in exon 19 and the same *TP53* mutation. After the start of thyrosine kinase inhibitor (TKI) therapy, a significant regression of the enlarged mediastinal lymph nodes was seen.

### Case 5

Case 5 was a 65 years old male with a centrally growing lung tumour engaging both the upper and lower right lobe and with radiologically suspected mediastinal invasion (i.e. T4). EBUS-guided fine needle aspirations detected a lymph node metastasis to position 12 only (N1). The cytology favored adenocarcinoma, and targeted NGS showed no treatment predictive mutations but a *BRAF* (c.1406G > T) and a *TP53* (c.375G > A) mutation. Neoadjuvant chemoradiotherapy induced a significant regression of the tumour, and a right sided pneumonectomy was performed where also a second subpleural tumour was discovered in the lower lobe. No lymph node metastases could be detected in the pulmonary or mediastinal lymph nodes.

**Fig. 5:**
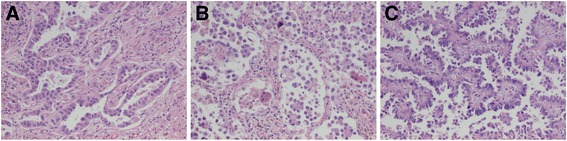
Case 5. Lung adenocarcinoma with intrapulmonary metastasis with different growth patterns but identical *BRAF* mutation. **a** The central tumour with acinary and lepidic (not shown) growth patterns. **b**-**c** The subpleural tumour with micropapillary and papillary growth patterns. All images HE and x10 objective

Both the central and the subpleural tumours were TTF-1 and napsin A positive non-mucinous adenocarcinomas. The central tumour, measuring at the most 5.3 cm, showed dense central fibrosis with focal microcalcifications constituting about 80% of the area, while the viable tumour was acinary apart from about 5% lepidic growth pattern. The subpleural tumour measured at the most 3.0 cm and exhibited micropapillary (60%) and papillary (40%) growth. STAS and focal microcalcifications were seen, but no fibrosis or necrosis. See Fig. 5.

Due to the difference in growth pattern between the tumours, both were analysed with targeted NGS. The previously noted *BRAF* mutation was detected in both tumours, while only the central tumour had a *TP53* mutation but not the same as in the previous cytological sample. See Table 1 for full NGS data. Based on the *BRAF* mutation it was concluded that the subpleural tumour was an intrapulmonary metastasis rather than a separate synchronous tumour.

One month after surgery the patient presented with an advanced intrathoracic infection but was successfully treated with intravenous and intrathoracic antibiotics. Two months after the surgery there was no sign of additional metastases or local relapse.

### Case 6

Case 6 was a 59 years old non-smoking woman who presented with a tumour in the right lower lobe, confirmed as an adenocarcinoma with a bronchial biopsy. Pyrosequencing did not show any *EGFR* mutation (any further analyses were not performed as the patient was planned for surgery). An additional suspicious area in the right upper lobe was detected on a CT scan before surgery, and the patient underwent a lobectomy of the lower lobe and a wedge resection of the upper right lobe. The upper lobe lesion was also an adenocarcinoma. Both tumours were non-mucinous, 4.0 cm (lower lobe) and 1.1 cm in size respectively. They were regarded similar in appearance and the upper lobe tumour was considered an intrapulmonary metastasis (pT4). No lymph node metastases were found and adjuvant chemotherapy was initiated.

After four months additional mutational analysis on the existing material was performed in order to investigate additional lines of treatment as there was radiological suspicion of more extensive tumour growth in the upper lobe than initially suspected. Pyrosequencing showed an *EGFR* mutation in the previously resected upper lobe tumour, while FISH for *ALK* gene rearrangements was negative (a later IHC staining for ALK was also negative). It was assumed that the *EGFR* mutation was also present in the lower lobe tumour, and that the first pyrosequencing analysis on the pre-surgical biopsy had failed to detect it, being a newly introduced method at the time. As the disease progressed, TKI was given for four months but with continued progression with spreading also to the left lower lobe. A core needle biopsy was taken from the latter to investigate if an *EGFR* resistance mutation had developed, but unexpectedly targeted NGS did not detect any *EGFR* mutation. See Table 1 for full NGS data.

**Fig. 6:**
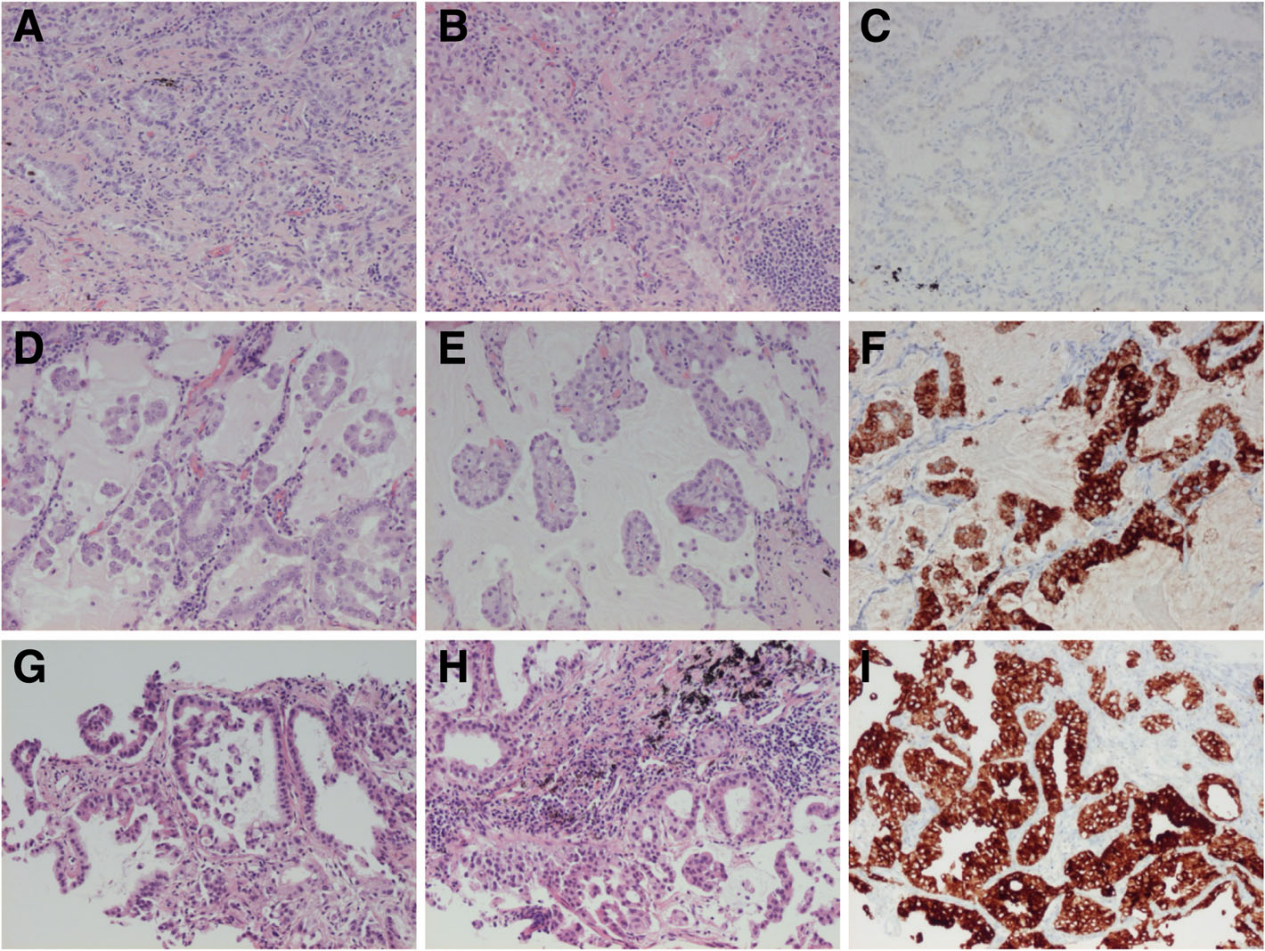
Case 6. Synchronous lung adenocarcinomas in right upper and lower lobes with different growth patterns and different genetic profiles with the contralateral metastasis from the latter. (**a**-**c**) The *EGFR* mutated right upper lobe tumour with acinary growth pattern. (**d**-**f**) The right lower lobe tumour with micropapillary (predominant), acinary and papillary growth patterns. (**g**-**i**) The metastasis to the left lower lobe. (**a**-**b**, **d**-**e**, **g**-**h**) HE, (**c**, **f**, **i**) ALK. All images x10 objective

In light of this, the tumours were again reviewed. The tumour in the right lower lobe had 75% micropapillary, 15% acinary, 5% papillary and 5% lepidic growth pattern and had a <5% mucinous component. The tumour in the right upper lobe had 100% acinary growth (<5% solid). Both were positive for TTF-1 and napsin A. The adenocarcinoma in the left lower lobe had predominant acinary but also micropapillary and lepidic growth on biopsy. See Fig. 6.

Targeted NGS did not detect any mutations in the tumours of the right lower lobe or the left lower lobe, while the right upper lobe tumour exhibited the previously known *EGFR* mutation and a *TP53* mutation. Based on the morphology and genetic profiles, it was concluded that the right upper and lower lobe tumours were synchronous adenocarcinomas and the left lower lobe tumour a contralateral metastasis from the *EGFR* negative right lower lobe tumour (explaining the lack of effect of TKI treatment). The patient was again put back on chemotherapy. However, after two months, the oncologist asked for PD-L1 analysis and noted that ALK analysis was missing from both the right lower lobe tumour and its left lower lobe metastasis. IHC staining for ALK proved to be positive in both tumours – see Fig. 6 – why treatment with a TKI for ALK was started.

## Discussion

In the present study we describe six cases where molecular genetic analysis was a valuable or necessary aid for accurate diagnosis. Two cases concerned metastases to the lungs and proving the relationship to the primary tumour when morphology and IHC staining was insufficient for definite diagnosis. The other four cases concerned synchronous primary lung cancers vs. intrapulmonary metastasis. As evident, single gene assays may be useful in some cases, but targeted NGS may be even more so, as seen especially in case 2 and 5.

Case 1 is an example of an atypical presentation of a common diagnosis. Colorectal cancer, a frequent cause of metastases to the lung, is commonly positive for CK20 and negative for CK7 [31]. Deviant CK7/CK20 pattern may be related to microsatellite instability, *BRAF* mutation and perianal Paget’s disease [32–35]. Positive TTF-1 (especially clone 8G7G3/1) is also very rare in colorectal cancer and such tumours still tend to be CK20+/CK7- [13, 15, 36–39]. Correct diagnosis is important as there is a difference in treatment and prognosis if a separate lung cancer with liver metastasis appears in a patient with previous rectal cancer instead of metastasis to the liver and lung from the rectal cancer. Although *KRAS* mutations are common in both colorectal and lung adenocarcinomas, [40] the finding of the same mutation in both tumours strongly supported that the pulmonary tumour was a metastasis. Although this case could have been solved with morphology only, the molecular genetic analysis was of aid for correct diagnosis.

Malignant adenomyoepithelioma in the breast, case 2, is a very rare diagnosis [41]. Epithelial-myoepithelial carcinomas are most often found in salivary glands, but may also derive from submucous glands in bronchi as a primary lung cancer. The diagnosis may be difficult, and although morphology and IHC staining is normally sufficient for the diagnosis, in the present case there were different opinions among experienced senior pathologists (who were all familiar with malignant adenomyoepithelioma) concerning the breast tumour before molecular genetic analysis. Although *PIK3CA* mutations (and especially c.3140A > G) are common in breast cancer, [40] the genetic profile strongly suggested that the lung tumours were metastases from the tumour in the breast. Although the IHC staining also supported the diagnosis malignant adenomyoepithelioma the molecular genetic analysis with targeted NGS was essential for diagnosis in this case.

Cases 3–6 are examples of multiple lung tumours where molecular genetic analysis was of aid in the differential diagnostics between synchronous primary lung cancer and intrapulmonary metastasis. Case 3 and 5 had different or partly different growth patterns but identical genetic profiles. In both cases, it did not really make a difference for treatment that stage pT4 could be established instead of the alternative pT3/pT2b plus a separate pT1. However, case 3 is also a good example that a tumour in a separate lobe may be a metastasis even in absence of lymph node involvement or distant metastasis. For example Klempner et al. has also previously shown that predominantly in situ (lepidic) growth does not rule out metastatic disease [42]. Case 4 is an example of adenocarcinomas with identical growth patterns located in different lobes but that were synchronous primary tumours. In case 5, the difference in growth pattern may of course be at least partly due to the neoadjuvant therapy. Case 6 had two adenocarcinomas that actually showed different growth patterns but was initially reported as having similar appearance, and optimal treatment could probably have been given should complete molecular analysis (with analysis of both *EGFR* and *ALK*) have been performed for both tumours right from the start.

Comprehensive histopathological subtyping, for adenocarcinomas preferably in 5% increments, with comparison of growth patterns and other morphological features, has been suggested as a tool for distinguishing synchronous/metachronous primary lung cancer from metastasis/relapse [1, 43]. However, non-mucinous lung adenocarcinomas with acinary, lepidic, papillary and/or solid growth are the most common, why subtyping may be of limited aid in many cases [44–46]. Also, most lung adenocarcinomas have the same IHC profile with positive CK7, TTF-1 and napsin A [1, 10]. Among the present cases, the results of IHC and growth patterns in 5% increments was not very helpful in cases 3–5, but was so in case 6.

A recent study by Schneider and co-workers showed a concordance between histological (Martini and Melamed criteria [19]) and molecular classification of synchronous primary lung cancers vs. intrapulmonary metastasis in 24 of 27 adenocarcinomas (89%) using a panel of seven genes (analysis with Sanger sequencing and FISH) [47]. In that study, two cases with different morphological features exhibited the same *KRAS* mutation (similar to our case 5) while one case with morphologically similar tumours had discordant *KRAS* mutation status suggesting separate synchronous primary tumours (like our case 4).

It is noteworthy that only selected cases were included in the present study. During the same period of time there were other cases at our department where molecular genetic analysis was not as helpful. For example, in one patient with previous colonic cancer three small suspected metastases to the lung were resected. One of the tumours was a metastasis of the colonic cancer, while two were non-mucinous lung adenocarcinomas with different growth patterns found in the same lobe. Targeted NGS revealed a *TP53* mutation in one of the lung cancers and no mutations in the other. Although a good concordance for *TP53* mutations has been reported between primary tumour and metastasis in some studies, a significant discordance has been shown in others (also our cases 4 and 5), making it a less reliable alteration for molecular genetic comparisons [29, 48–51]. The presence of *TP53* mutations in normal lung tissue of lung cancer patients supports its role as an early driver in lung cancer, [52] and it would be of interest to investigate if some *TP53* mutations more than others are prone to arise early in the process and be concordant between primary tumours and metastases.

In the literature, there are several studies on synchronous primary lung cancer vs. intrapulmonary metastasis with variable and rather limited number of included patients and with different molecular methods used. For example, in three rather large studies, Girard and co-workers used comparative genomic hybridization for analysis of copy number variation, Warth and co-workers used Sanger sequencing and loss of heterozygosity, while Yatabe and co-workers used RT-PCR [21, 28, 53]. According to comprehensive reviews a generally high but variable (61-100%) concordance for driver mutations (*EGFR* and *KRAS*) between primary tumours and metastases has been reported for lung cancer [30, 54]. It has been discussed that discordance of molecular genetic profiles between spatially or temporally different tumours with common origin may partly be due to methodological issues [30, 54–56]. In recent well-performed studies using targeted NGS, Vignot and co-workers found no discordance in driver mutations between primary lung cancers and their metastases, while Goswami and co-workers found a few cases of colonic cancer where a pathogenic *KRAS* mutation was not found in distant metastases [49, 50]. However, Pelosi and co-workers found significant intra-tumour heterogeneity including co-existence of *EGFR* and *KRAS* mutations in minority clones using laser microdissection of multiple areas in lung adenocarcinomas [57].

One problem for studies on multiple lung tumours is the lack of diagnostic gold standard. If histopathological features with/without addition of radiological findings are used as basis, it may lead to wrong conclusions about molecular concordance and discordance between primary tumours and metastases. But then again, there is insufficient knowledge when or how often the molecular genetic profile can be used as the sole diagnostic tool to determine if two tumours are related or not. Liu and co-workers argued that a case with three lung cancers, all with the same *EGFR* mutation, still were likely to be synchronous tumours based on about 40 discordant non-driver mutations found with WES [58]. However, given the number of mutations typically found with WES in lung cancers, [26] it is difficult to be certain, and WES is maybe not more helpful than targeted sequencing for comparison of molecular genetic profiles in these situations.

In the IASLC lung cancer staging project for the upcoming TNM8 it has been proposed to consider multiple tumours as from a single tumor source if exactly matching breakpoints are identified using comparative genomic hybridization [56] (although alternative techniques for breakpoint analysis is also acknowledged [59]). Different or same biomarker pattern (e.g. driver mutations) is considered to be relative arguments that favour separate or single tumour source, respectively. We agree it is difficult to draw certain conclusions given the evidence of today, though in our opinion, different driver mutations (such as *EGFR* or *KRAS* mutations or *ALK* or *ROS1* rearrangements, like in our cases 4 and 6) probably quite strongly support synchronous/metachronous tumours. According to the IASLC proposals, multiple lesions of adenocarcinoma in situ, minimally invasive adenocarcinoma or lepidic predominant adenocarcinoma should be regarded as separate synchronous tumours, [60] supported by highly discordant mutational profiles, also for driver mutations [61].

The number of cases with multiple tumours in the lung or with a lung tumour where the relationship to a tumour in another organ cannot be established with morphology and IHC staining (squamous and neuroendocrine cancers excepted) is quite limited. However, at the present, it seems reasonable to perform treatment predictive molecular analysis on all tumours in cases with multiple lung nodules (at least if there are no nodal or distant metastases) and also in cases where the relationship between two tumours (including tumours outside the lung) is unclear. Consequently, pathology departments handling these cases need competence in molecular pathology (including awareness of the possibilities and pitfalls of molecular pathology for diagnostics in patients with multiple tumours) and continue to introduce techniques that may be helpful in these situations. Although targeted NGS and other elaborate techniques cost more than single-gene analyses, the price is still low compared to the cost of e.g. treatment with TKI, and there is possible gain also for treatment prediction. A more important limitation is turnaround time, which is often more important than extreme sensitivity in daily health care.

For the future, there is a continuing need for relatively large studies on heterogeneity within a single tumour and between primary tumour and obvious metastasis (such as lymph node or brain metastasis). The diagnostic and clinical importance of minority clones should be further examined. Which genetic changes are stable from primary tumour to metastases and what techniques are best at discovering them are both of great interest. For example, the potential of single cell analyses (e.g. PCR or NGS) should be further explored in solid tumours [62]. If comparison of molecular genetic profile alone or together with histopathological features and/or clinic-radiological input is the best for diagnostics of multiple lung tumours also needs to be further investigated.

## Conclusions

Comparison of molecular genetic profile may be an important tool for determination of relationship between tumours, at least in some cases. Such comparisons should always be considered in unclear cases and international guidelines should preferably stress its role in differential diagnostics of intrapulmonary metastasis vs. synchronous primary lung cancers. Further studies on concordance and discordance of molecular genetic profiles between spatially or temporally different tumours with common origin and which analyses are best for diagnostics of such cases may be helpful for improved handling of patients with pulmonary tumours.

## Abbreviations

ACCP: American College of Chest Physicians
CK: Cytokeratin
CT: Computed tomography
EBUS: Endobronchial ultrasound
EGFR: Epidermal growth factor receptor
FFPE: Formalin-fixed paraffin-embedded
FISH: Fluorescence in situ hybridization
HE: Hematoxylin-eosin
IHC: Immunohistochemical
NGS: Next-generation sequencing
STAS: Spreading through air spaces
TKI: Thyrosine kinase inhibitor
TTF-1: Thyroid transcription factor 1
WES: Whole exome sequencing
